# An Insight into the Flammability of Some Bio-Based Polyesters

**DOI:** 10.3390/polym9120706

**Published:** 2017-12-12

**Authors:** Loïc Dumazert, Rodolphe Sonnier

**Affiliations:** Ecole des Mines d’ Alès, Centre des Matériaux des Mines d’ Alès—Pôle Matériaux Polymères Avancés, 6 Avenue de Clavières, 30319 Alès CEDEX, France; loic.dumazert@mines-ales.fr

**Keywords:** polyesters, poly(3-hydroxybutyrate), flammability, pyrolysis, pyrolysis-combustion flow calorimeter

## Abstract

The heat release capacity of polymers can be generally predicted using a method based on the additivity of group contributions (the Van Krevelen approach). Nevertheless, there are some exceptions, evidencing that this approach is insufficient and must be completed. In this study, the kinetic triplet accounting for the description of pyrolysis is identified for 11 polymers. Activation energy and the frequency factor are calculated using Kissinger’s method. Reaction models are chosen among the Avrami–Erofeev functions. The high flammability of poly(3-hydroxybutyrate) and the underestimation of its heat release capacity using the Van Krevelen approach are explained from these parameters. The results highlight the possibility of improving the model, using additional but easily accessible data.

## 1. Introduction

**Figure 1:**
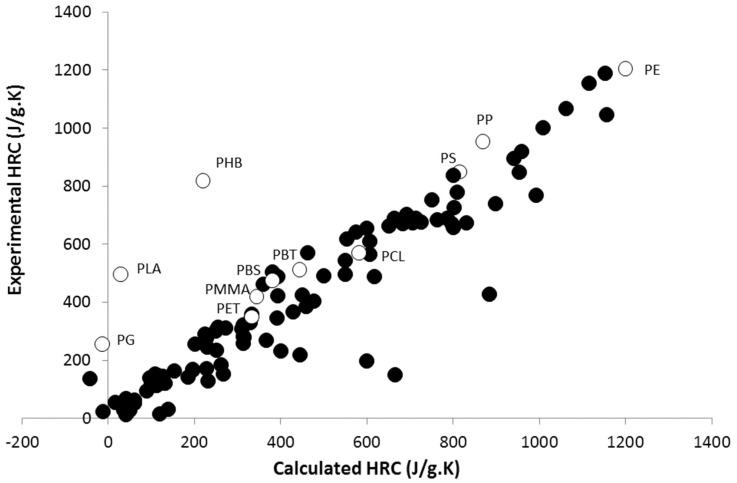
Experimental versus calculated heat release capacity (HRC) for more than 100 polymers (data drawn from [4]). White circles correspond to the 11 polymers studied in this work.

Recently, methods based on the Van Krevelen approach have been proposed to assess the flammability of a polymer at microscale, using a pyrolysis-combustion flow calorimeter (PCFC) [1,2,3,4,5]. A PCFC is an apparatus that allows the heating of a few milligrams of a polymer to 750 °C, according to a linear heating rate (1 K/s) under nitrogen. Pyrolytic gases are continuously sent to a combustor at 900 °C in an excess of oxygen, and combustion is completed. The amount of heat released is calculated according to the oxygen depletion method, allowing some important flammability properties to be measured, such as Heat Release Capacity (HRC) or Total Heat Release (THR). HRC is the ratio between the peak of Heat Release Rate (pHRR) and the heating rate (typically 1 K/s). The pHRR is one of the main parameters used to assess the flammability of a polymer. THR corresponds to the area under the Heat Release Rate (HRR) curve. In previous works [4,5], the contributions to HRC and THR were calculated for more than 45 chemical groups, and the properties of around 130 polymers were predicted with good accuracy (Figure 1).

**Figure 2:**
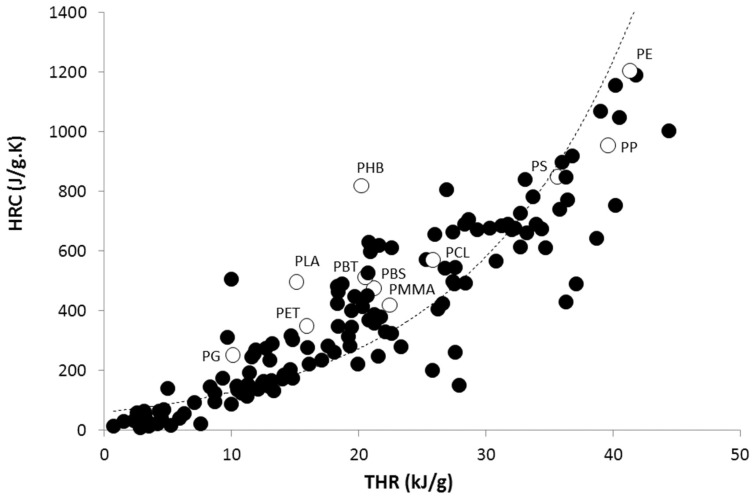
Correlation between heat release capacity and total heat release (THR) measured in a pyrolysis-combustion flow calorimeter (PCFC) for around 150 pure polymers (data drawn from [4]). White circles correspond to the 11 polymers studied in this work.

Unfortunately, some predictions failed, in particular for several aliphatic polyesters, including polyglycolide (PG), poly(lactic acid) (PLA), and poly(3-hydroxybutyrate) (PHB). These three polymers exhibit experimental HRCs much higher than the calculated ones. This failure may be partly due to the limits of the method for PG and PLA. Indeed, the negative contributions assigned to the ester groups become irrelevant when the ester mass fraction in the polymer is too high. Nevertheless, PHB has the same ester fraction as poly(butylene succinate) (PBS), but its HRC is much higher than for PBS and other polyesters (Figure 2).

The reason behind this failure is obviously related to specific pyrolysis mechanisms. Indeed, HRC depends on the heat of complete combustion (Δ*h*) and pyrolysis kinetics parameters, i.e., the so-called kinetic triplet (*E*_a_, *A*, *f*(α)). This kinetic triplet (*E*_a_, *A*, *f*(α)) is used to adequately describe pyrolysis kinetics through Equation (1):

(1) $$\frac{d\alpha }{dt}=Ae^{\frac{-E_{a}}{RT}}f\left(\alpha \right)$$

in the equation, α is the extent of the reactant conversion (i.e., the reacted fraction), *R* is the gas constant, *E*_a_ is the activation energy, *A* is the frequency (or pre-exponential) factor, and *f*(α) is the reaction model (there are many reaction models available [6,7]).

In order to understand why some predictions failed, and to improve our model, the knowledge of the triplet for each polymer would be useful, even if the physical meaning of the kinetic triplets is controversial, for a couple of reasons comprehensively discussed in the literature [6].

There are many articles dealing with the pyrolysis mechanisms, in great detail, for almost all polymers. Several authors have already studied the pyrolysis of PHB in great detail [8,9,10,11], and a step of auto-catalytic degradation was reported [9]. Nevertheless, the quantitative data (namely, the kinetic triplet to describe the pyrolysis) vary greatly from one study to another. Even the activation energy must be considered only as apparent, and its value for a same polymer may vary to a large extent. As an example, Ariffin et al. have listed the activation energy values for PHB obtained by various research teams [9]. The activation energy ranges from 110 to 380 kJ/mol, according to the authors and the heating rate methods. The same discrepancy can be found for PLA or polycaprolactone (PCL), for example. From a practical point of view, it is hardly possible to use these data for an initial quick assessment of the thermal stability and flammability of polymers, only based on their chemical structure.

The objective of this work is to use a simple and standardized procedure to identify a kinetic triplet, allowing for the correct calculation (i.e., with good accuracy) of the temperature and the intensity of the pHRR, measured in a PCFC, even if the whole HRR curve is not perfectly described. Our motivation is purely to improve the model previously proposed, based on the additivity of group contributions. More specifically, we would like to identify why PHB but also PLA and PG exhibit surprisingly high HRC values, from the knowledge on their kinetic triplets. The procedure is based on Kissinger’s method for calculating the activation energy and the frequency factor, and considers only the Avrami-Erofeev functions as reaction models. No knowledge of the precise pyrolysis mechanisms is required. The first results on a set of 11 polymers show that the high HRC measured for PHB is highlighted well by the calculated kinetic triplet.

## 2. Methodology

### 2.1. Materials

The commercial polymers used in this study are listed in Table 1. Seven of them are polyesters. The four remaining ones are commonly used polymers.

### 2.2. Experiments

The thermal stability of the samples was determined using thermogravimetric analysis (TGA Pyris 1, Perkin-Elmer, Waltham, MA, USA). The sample weights were approximately 5 mg. Experiments were performed between 30 and 750 °C at different heating rates (5, 10, 20, and 40 K/min) under nitrogen (20 mL/min). The decomposition temperature used with Kissinger’s method was determined as the temperature of the peak of mass loss rate on a dTG (derivative thermogravimetric) curve.

The flammability of the polymers listed in Table 1 was analyzed using a PCFC (FTT, Derby, UK) under standard conditions, i.e., anaerobic pyrolysis from 25 to 750 °C at 1 °C/s in nitrogen, and complete combustion in an excess of oxygen at 900 °C [12].

### 2.3. Method

The objective of this work is to compare the kinetic triplets of an extended set of polymers, in order to understand why some aliphatic polyesters exhibit a higher pHRR than expected. The description of pyrolysis mechanisms is out of the scope of this work. A purely mathematical description of the pyrolysis correctly predicting the HRR is the main task.

HRR is closely related to mass loss rate (MLR) through Equation (2):

HRR = MLR × Δ*h* (2)

here, Δ*h* represents the heat of complete combustion (in the case of a PCFC, the combustion is complete). The heat of complete combustion is measured using a PCFC in standard conditions. For the studied polymers, it is reasonable to consider that this value remains constant throughout the whole pyrolysis process. The measured values are listed in Table 1. HRR and Δ*h* are calculated in W/g and J/g respectively, therefore MLR is calculated in $\frac{g/g}{s}=s^{-1}$.

Both in TGA and in a PCFC, the sample is heated using a constant heating rate under nitrogen flow. In theory, both devices allow the calculation of the same kinetic triplet using the same methods. However, the ability to modify the heating rate is more limited in a PCFC, and the accuracy of the temperature is lower. Therefore, we decided to first determine the activation energy and the frequency factor using TGA.

In the first step, TGA was carried out to calculate the activation energy of pyrolysis, through a procedure involving multiple heating rates. Two methods were used and compared: the Flynn-Wall-Ozawa method and Kissinger’s method. Details about these methods can be found elsewhere [13,14,15]. The latter also allows the calculation of the frequency factor. TGA provides inconsistent data at the highest heating rates, for unknown reasons, especially in the case of polyglycolide. Therefore, for that polymer, the selected heating rates were 2, 5, and 10 K/min.

The MLR curve in a PCFC can be deduced from the HRR curve using Equation (2). From this curve, and the knowledge of *E*_a_ and *A*, a reaction model *f*(α) can be identified to determine the whole kinetic triplet (*E*_a_, *A*, *f*(α)). The interpretation of *f*(α) as a reaction mechanism is probably irrelevant in many cases [6]. Some authors have used a combination of reaction models to accurately fit the TGA curves [16]. Our approach was different and purely practical. Only first-order reaction and Avrami-Erofeev models were considered. A reasonably good fit was obtained using these models for all polymers, as shown in the following. Moreover, this choice allowed for comparing the reaction models of the different polymers through only one parameter (*n*). The general form of the chosen models is shown in Equation (3):

(3) $$f\left(\alpha \right)=n\left(1-\alpha \right){\left(-Ln\left(1-\alpha \right)\right)}^{1-\frac{1}{n}}$$

in this equation, *n* ranges between 1 and 4 [17]. Corresponding to the first-order reaction, *n* = 1, and the other cases correspond to Avrami–Erofeev models.

If the triplet (*E*_a_, *A* and *f*(α)) is correctly identified, then the MLR curve is well-fitted, i.e., the HRR curve can be rebuilt by multiplying the calculated MLR by the heat of complete combustion (Equation (2)).

Note that the heating rate in a PCFC is 1 K/s (60 K/min). Therefore, the activation energy and the frequency factor calculated using TGA are used to extrapolate the pyrolysis kinetics outside the temperature experimental range, as recommended by Vyazokin [6]. However, we have found a slight but systematic shift of activation energy. Indeed, the activation energy *E*_a(PCFC)_, used to simulate the mass loss rate at 1 K/s (i.e., to simulate in fine the HRR curve measured in PCFC), is slightly lower than the activation energy *E*_a(TGA)_, calculated by Kissinger’s method from TGA data ($\frac{E_{a\left(\mathrm{PCFC}\right)}}{E_{a\left(\mathrm{TGA}\right)}}\approx 0.95).$ The comparison is shown in the supporting information (Figure S1).

## 3. Results and Discussion

### 3.1. Activation Energy of Pyrolysis

Table 2 presents the activation energies calculated from the Flynn-Wall-Ozawa and Kissinger methods. The activation energy using the Flynn-Wall-Ozawa method was calculated only for the conversion range 0.2–0.8. The values for lower or higher conversions are often less reliable. The values for activation energy are highly constant over the whole conversion range for some polymers (PE (polyethylene), PP (polypropylene), PHB (poly(3-hydroxybutyrate), PG (polyglycolide), and PS (polystyrene)). For other polymers, the activation energy tends to increase when conversion increases. Both cases are common. For example, Aoyagi et al. have observed that apparent activation energies for PCL and PHB remain constant, while activation energy for PLA increases from 80 to 160 kJ/mol [8].

**Figure 3:**
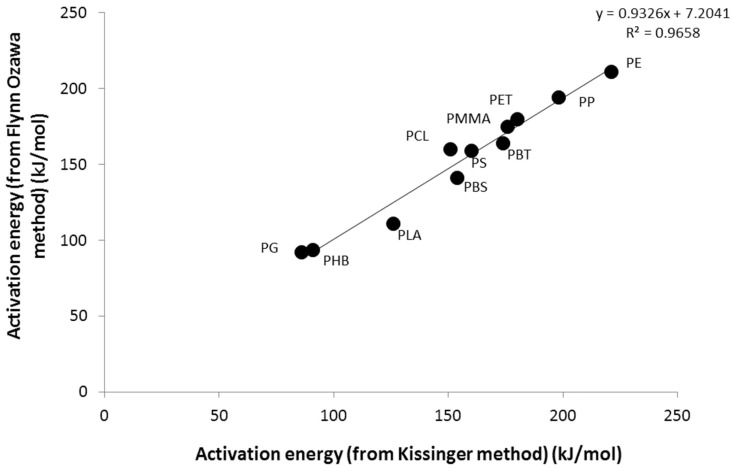
Comparison of activation energy values obtained from the Kissinger and Flynn-Wall-Ozawa methods.

Figure 3 shows the agreement between the activation energies measured using both methods (the mean activation energy for the conversion range 0.2–0.8 was used for the Flynn-Wall-Ozawa method).

As explained in the introduction, the activation energy values in the literature are not consistent, and the reliability of the comparison of values from different works is limited. However, it is notable that the values obtained in this work are in quite good agreement with those from some other reports that used the same methods. For example, activation energies between 100 and 120 kJ/mol for PHB (slightly higher than the values found in our work) have been measured using multiple heating rates [8,9]. Table S1 in the supporting information gives an overview of the activation energies found in literature.

**Figure 4:**
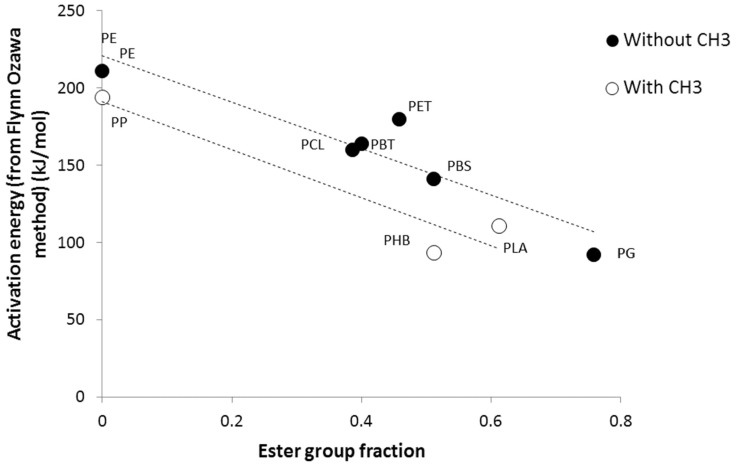
Activation energy (from the Flynn-Wall-Ozawa method) versus ester group fraction/ for polymers with or without a CH_3_ group.

Figure 4 illustrates how the ester group fraction or the presence of a CH_3_ pendant group on the polymer backbone influences the activation energy value. The activation energy calculated from the Flynn-Wall-Ozawa method decreases when increasing the fraction of the ester group. For CH_3_-free polymers, the activation energy decreases linearly from 211 kJ/mol for PE (ester fraction 0) to 92 kJ/mol for polyglycolide (ester fraction 0.76). A similar decrease is observed for polymers containing a CH_3_ group, but whose activation energy is systematically lower for a given ester fraction. For example, the activation energy is 194 kJ/mol for PP (versus 211 kJ/mol for PE) and 91 kJ/mol for PHB (versus 141 kJ/mol for PBS). For these last two polymers, the ester fraction is 0.51.

Note that the dependence of activation energy measured using the Kissinger method with the presence of a CH_3_ group is less obvious, even if both methods (Flynn-Wall-Ozawa and Kissinger) provide well-correlated results.

**Figure 5:**
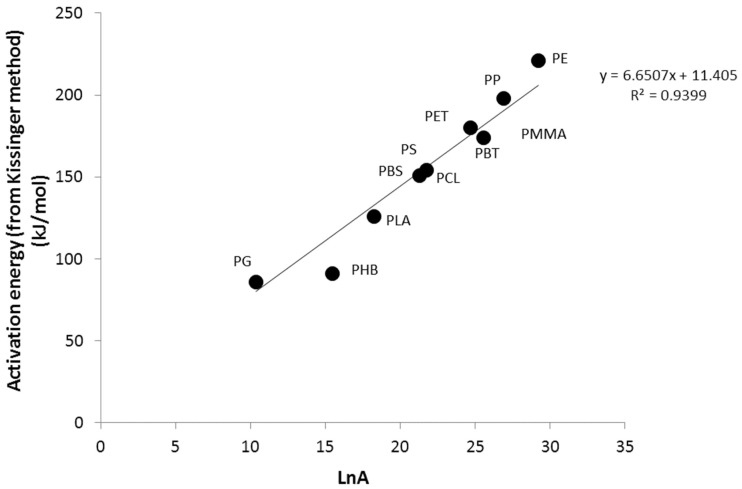
Activation energy versus *LnA* from the Kissinger method. The straight line is calculated considering all polymers except poly(3-hydroxybutyrate) (PHB).

It is well known that the frequency factor and the activation energy are often correlated (compensation effect) [6]. This typical relation is also observed with our results (Figure 5). However, the pair (*E*_a_, *A*) is offset from the straight line for PHB. The frequency factor is higher than expected. A higher frequency factor leads to a faster degradation, all other factors being equal.

### 3.2. Calculation of Activation Energies from Chemical Structure

The Van Krevelen approach has been used to predict a huge number of polymer properties, including those related to thermal degradation [18]. To the best of our knowledge, this method has never been used to calculate the activation energy for pyrolysis. There are other, more refined methods for assessing the activation energy, but they need knowledge of the pyrolysis pathway. Ariffin et al. give an example of the calculation of β-elimination in PHB, using the molecular orbital calculation method [9]. The present section is an attempt to show that the frequency factor and the activation energy may be well-predicted from the contributions of only four chemical groups. The activation energy *E*_a_ (respectively *LnA*) is calculated from the contributions of *E*_ai_ (respectively *LnA*_i_) to the activation energy of the chemical groups, as well as their molar masses *M*_wi_ (Equations (4) and (5)).

(4) $$E_{a}=\frac{\sum_{i}M_{\mathrm{wi}}E_{\mathrm{ai}}}{\sum_{i}M_{\mathrm{wi}}}$$

(5) $$LnA=\frac{\sum_{i}M_{\mathrm{wi}}LnA_{i}}{\sum_{i}M_{\mathrm{wi}}}$$

Frequency factor and activation energy are both calculated using the Kissinger method for nine polymers among the eleven studied ones. Table 3 lists the contributions of the four chemical groups present in these nine polymers. PS and PMMA (poly(methyl methacrylate)) were not included in this calculation, because they contain additional chemical groups.

**Figure 6:**
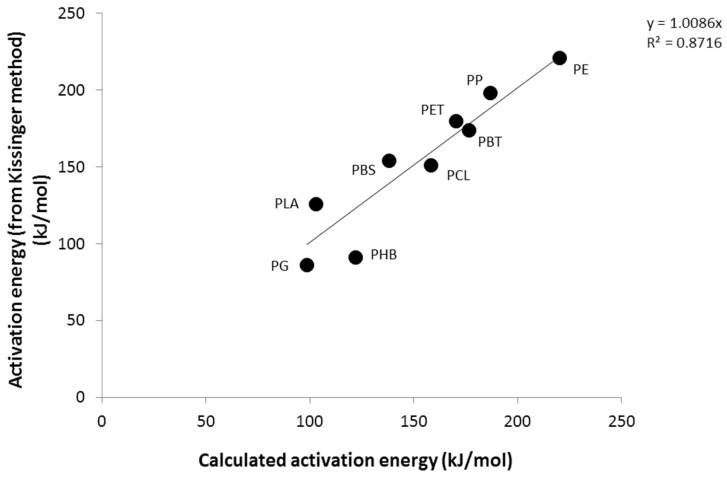
Experimental versus calculated activation energies for the studied polymers.

**Figure 7:**
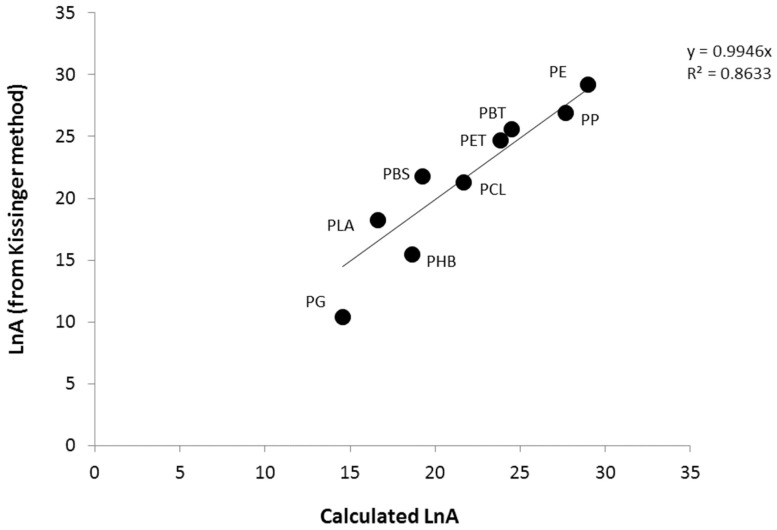
Experimental versus calculated *LnA* for the studied polymers.

Figure 6 and Figure 7 show the correlation between the experimental values (i.e., obtained from the Kissinger method) and the calculated values, using values in Table 3. The correlation is quite good between the activation energy and the frequency factor. Note that the discrepancy between the experimental data and the calculated frequency factor concerns mainly the PHB, but also the PG (for the frequency factor). The contributions to activation energy and frequency factor are logically correlated (see Figure S3 in Supplementary Materials).

## 4. Reaction Model

As explained above, the MLR curve in a PCFC is deduced from an HRR curve. For each polymer, we used the activation energy and frequency factor already calculated, as well as the parameter *n* listed in Table 4, to fit this MLR curve. Note that the activation energy has been slightly modified, as explained in the section “Method”. The reaction models (Equation (3), i.e., Avrami-Erofeev models) for the various polymers differ only from the parameter *n*. Note the values listed in Table 4 are also the best ones to fit the TGA curves (except PP, for which the *n* value may be closer to 2). In other words, the kinetic triplet used to fit MLR curves obtained from PCFC is fully defined from TGA.

**Figure 8:**
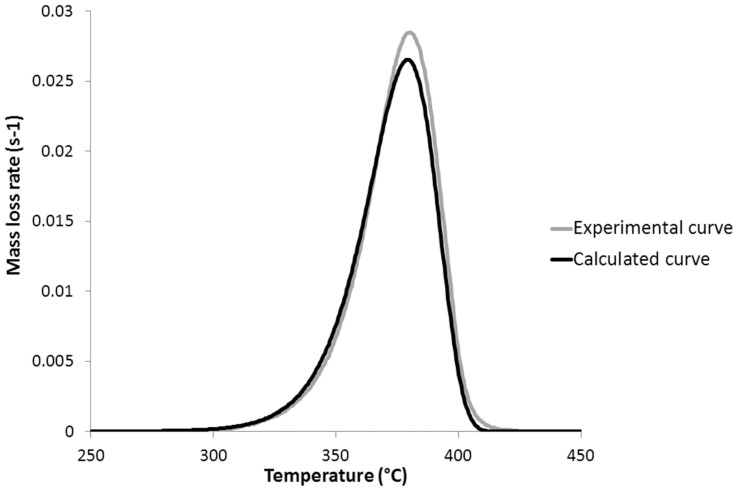
Experimental and calculated mass loss rate curves for poly(lactic acid) (PLA) at 1 K/s.

**Figure 9:**
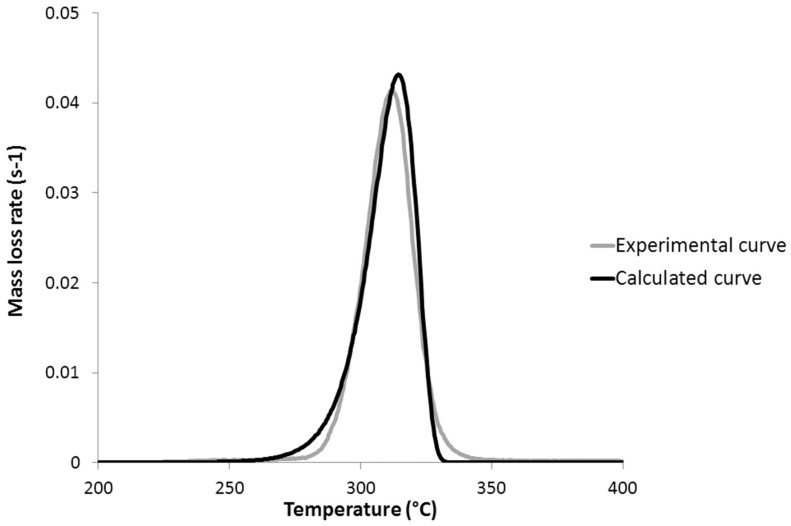
Experimental and calculated mass loss rate curves for PHB at 1 K/s.

Figure 8 and Figure 9 show the experimental and calculated mass loss rate curves for PLA and PHB, respectively. Experimental curves have been obtained from HRR curves in a PCFC, using Equation (2). The curves for other studied polymers can be found in the Supplementary Materials. It is noticeable that the reaction models fit the mass loss rate curves quite well, at least when the mass loss rate is moderate to high (>0.005–0.01 s^−1^). The temperature and the intensity of the peak of mass loss rate are both well-fitted. The accuracy of the experimental curve is lower at low and high conversions, in particular for PHB. The calculated MLR curve for PCL is less satisfying, but the temperature and the intensity of the peak of the MLR remain well-fitted.

**Figure 10:**
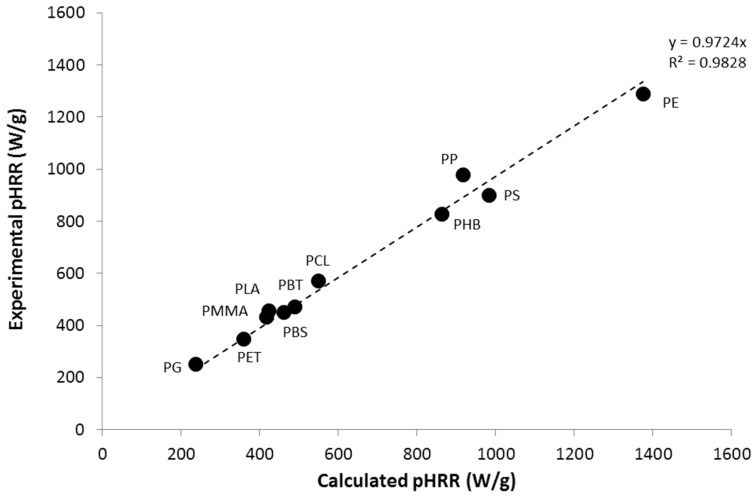
Comparison between the calculated and experimental peak of Heat Release Rate (pHRR) for the 11 studied polymers.

Using Equation (2), and the value of the heat of complete combustion listed in Table 1, it is easy to rebuild the HRR curves, and to compare the calculated and experimental pHRR. Figure 10 shows the good agreement between the pHRRs calculated using the kinetic triplets determined previously and the experimental pHRR.

It is quite surprising that such a result was obtained for only one type of reaction model (the Avrami-Erofeev model), which allows for an easy comparison between polymers. The simple approach developed here (based on Kissinger’s method and the Avrami-Erofeev model) should be extended to other polymers, in order to check its applicability. The main interest of this approach is to allow an easy comparison between polymers, as well as the identification of polymers for which pyrolysis kinetics appears unexpected and needs further research. The last section illustrates the usefulness of the method.

## 5. Discussion about the Flammability of PHB

The above comparison of kinetic triplets allows for establishing some relationships between the chemical structure of polymers and some parameters. In particular, the influence of an ester group on the polymer backbone, or the presence of a methyl group as a pendant group is highlighted. Both cases contribute to lowering the activation energy, which leads to a low thermal stability. PG and PLA exhibit low activation energy due to a high ester fraction. PHB has the same ester fraction as PBS, but a lower activation energy due to the presence of a CH_3_ pendant group.

**Figure 11:**
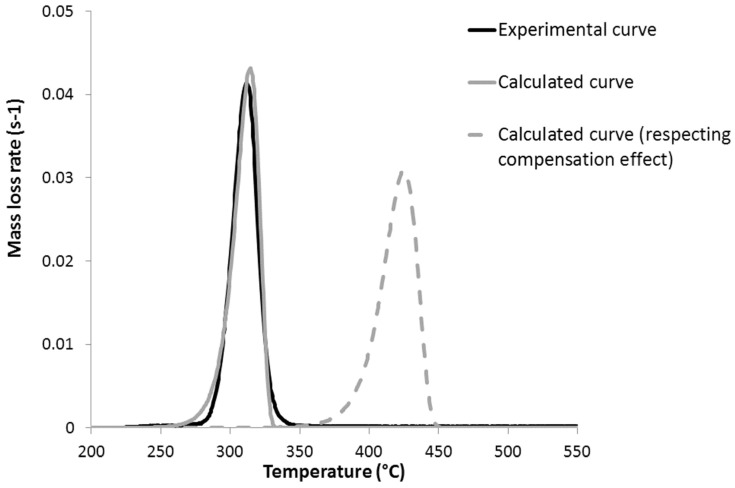
Experimental and calculated mass loss rate curves at 1 K/s for PHB (in a PCFC). Calculation was done with and without respecting the compensation effect.

Nevertheless, due to the compensation effect, the frequency factor decreases together with activation energy. Therefore, the influence of activation energy on thermal stability is real, but limited. However, for PHB, the frequency factor is higher than expected, promoting a faster decomposition. Figure 11 shows the MLR curve of PHB if the compensation effect was respected, i.e., if the frequency factor was correlated with the activation energy through the relationship in Figure 5. In that case, the peak of MLR would be lower and shifted to higher temperature. The reason PHB does not respect the compensating effect is unknown.

**Figure 12:**
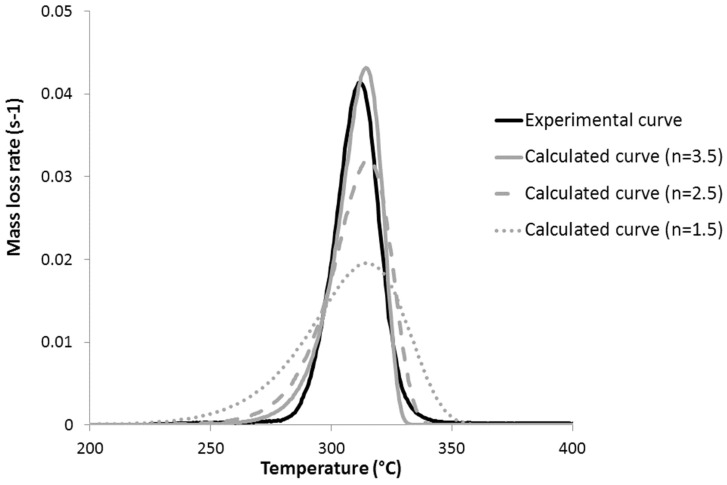
Experimental and calculated mass loss rate curves at 1 K/s (in a PCFC) with different values for *n* for PHB.

Finally, PG, PLA, and PHB also exhibit a high value for parameter *n* (2–2.5 and even 3.5 for PHB versus 1.5 for all other polyesters). A high value for *n* is also responsible for an increase of the pyrolysis rate. Figure 12 shows the calculated mass loss rate curves of PHB for different values of *n*. An increase in the *n* value does not influence the temperature of the peak, but increases significantly its intensity (i.e., also the intensity of pHRR).

Note that these two sources of discrepancy for PHB (higher frequency factor than expected and high *n* value) are enough to explain why its HRC is so high. When considering a value for *n* = 1.5 and respecting the compensation effect, the HRC is only 380 J/(g·K). This value is close to the dotted line in Figure 2, and in agreement with the calculated value using a Van Krevelen method (Figure 1). In the case of PLA and PG, the calculation with *n* = 1.5 (versus 2 and 2.5 respectively) leads to an HRC equal to 320 and 145 J/(g·K) respectively. These values are also close to those expected from the rough correlation between HRC and THR seen in Figure 2.

## 6. Conclusions

The originality of this work is obviously not the use of very classical methods to calculate the kinetic triplet. However, the procedure described here should allow the main flammability properties of polymers to be predicted more accurately.

Indeed, the contribution to the HRC of ester functions fails to predict the HRC of all aliphatic polyesters on its own. On the other hand, taking into account the kinetic triplet—Activation energy, frequency factor, and the *n* parameter (from the Avrami-Erofeev model)—Accounts for the surprising behavior of PHB. Indeed, this polymer is the main exception, for which the predicted HRC was much lower than the experimental value.

Moreover, it may be possible to calculate the activation energy using the Van Krevelen method, based on the additivity of group contributions. Calculating the contributions of chemical groups to activation energy, as well as establishing the relations between the chemical structure and the values of the frequency factor and *n* parameter, are the next steps to improve the model.

Of course, such a Van Krevelen method can appear to be too simplistic. Moreover, the used parameters are a purely mathematical description, and probably have no physical meaning. Therefore, there are obvious limits for this approach. However, the approach could be powerful if it allows the flammability of polymers to be assessed quickly, and with good accuracy.

## Figures and Tables

**Table 1:** Polymers studied in this work.

Polymer	Manufacturer	Chemical structure	Heat of complete combustion (kJ/g)
(High density) Polyethylene	Eraclene FC 92 Polimeri Europa	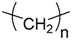	42
Polypropylene	3100 MT3 1P2381 Atofina	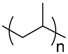	39
Polyethylene terephthalate	Arnite D04 300	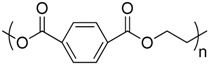	16
Polybutylene terephthalate	Valox 325F GE Plastics	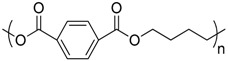	20
Polycaprolactone	CAPA 6800 Perstorp	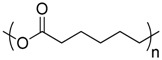	26.5
Polybutylene succinate	2003F Xinfu Pharm	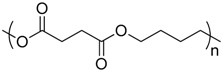	21.5
Poly(3-hydroxybutyrate)	Biocycle 1000	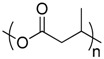	20
Polylactic acid	Unitika 6201-D	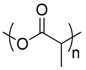	16
Polyglycolide	Sigma Aldrich	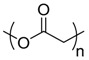	10.5
Polymethyl methacrylate	Altuglas V825T	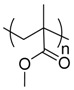	23
Polystyrene	Lacqrène 1340 Atofina	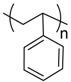	35

**Table 2:** Kinetic parameters measured for all studied polymers.

Polymer	Flynn-Wall-Ozawa method					Kissinger method	
	*E*_a_ (kJ/mol)					*E*_a_ (kJ/mol)	*LnA*
	α = 0.2	α = 0.4	α = 0.6	α = 0.8	Mean		
PE	210	217	210	209	211	221	29.2
PP	202	194	192	187	194	198	26.9
PET	169	177	182	193	180	180	24.7
PBT	157	163	165	172	164	174	25.6
PCL	139	163	166	172	160	151	21.3
PBS	125	140	148	151	141	154	21.8
PHB	98	93	89	84	91	91	15.4
PLA	109	100	116	120	111	126	18.2
PG	95	89	91	94	92	86	10.4
PMMA	164	170	174	193	175	176	27.4
PS	159	158	159	162	159	160	22.0

**Table 3:** Contributions to the activation energy and frequency factor for four chemical groups.

Chemical groups	*M*_w_ (g/mol)	Contribution to	
		*E*_a_ (kJ/mol)	*LnA*
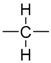	14	220	29
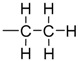	29	170	27
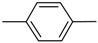	76	280	38
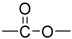	44	60	10

**Table 4:** Values of the parameter *n* for all studied polymers.

Polymer	*n*
PE	2
PP	1.5
PET	1.5
PBT	1.5
PCL	1.5
PBS	1.5
PHB	3.5
PLA	2
PG	2.5
PMMA	1
PS	2

## Supplementary Materials

The following are available online at www.mdpi.com/2073-4360/9/12/706, Figure S1: Comparison between activation energies calculated from thermogravimetric analysis (TGA) data using Kissinger’s method and activation energies used to fit the pyrolysis-combustion flow calorimeter (PCFC) results; Figure S2: Correlation between the contributions to activation energy and frequency factor; Figure S3: Experimental versus calculated mass loss rate curves for PE at 1 K/s; Figure S4: Experimental versus calculated mass loss rate curves for PP at 1 K/s; Figure S5: Experimental versus calculated mass loss rate curves for PET at 1 K/s; Figure S6: Experimental versus calculated mass loss rate curves for PBT at 1 K/s; Figure S7: Experimental versus calculated mass loss rate curves for PBS at 1 K/s; Figure S8: Experimental versus calculated mass loss rate curves for PCL at 1 K/s; Figure S9: Experimental versus calculated mass loss rate curves for PG at 1 K/s; Figure S10: Experimental versus calculated mass loss rate curves for PS at 1 K/s; Figure S11: Experimental versus calculated mass loss rate curves for PMMA at 1 K/s; Table S1: Activation energies from literature for studied polymers.
