# Supplementary material for: An Insight into the Flammability of Some Bio-Based Polyesters
Source: Polymers (Basel). 2017 Dec 12;9(12):706. doi: 10.3390/polym9120706 (PMC6418667; doi:10.3390/polym9120706)
Supplement: Supplementary file 1 [file polymers-09-00706-s001.pdf]

Supporting information

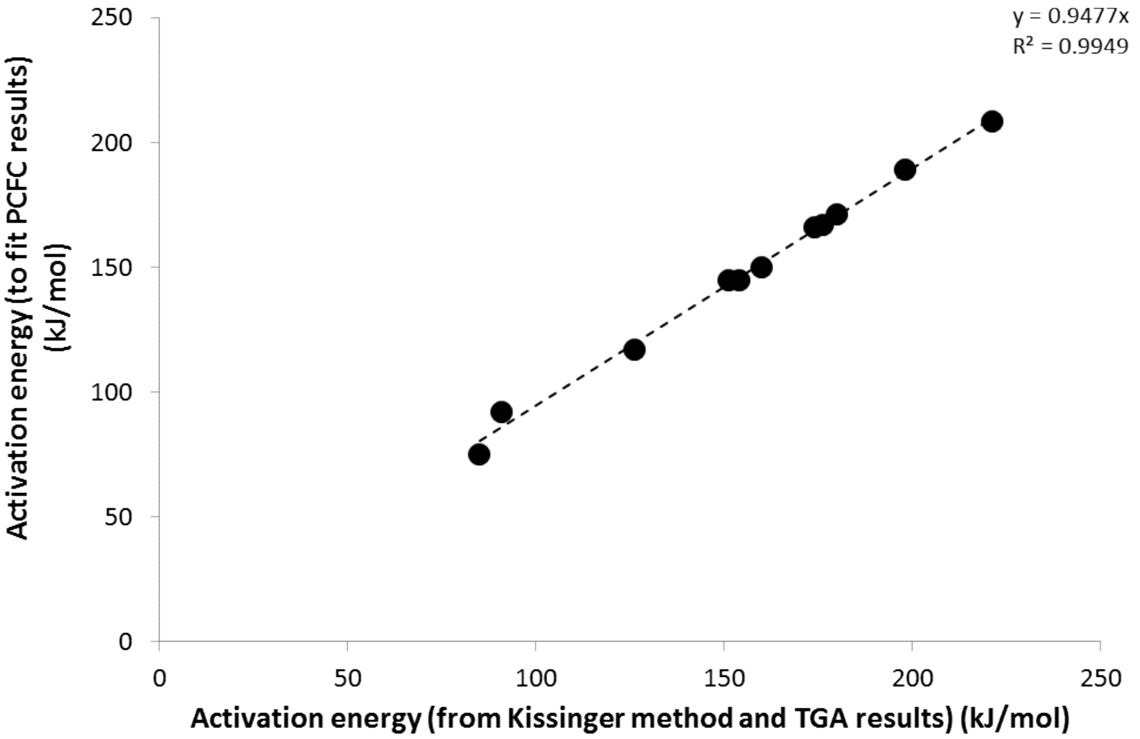

Figure S1 – Comparison between activation energies calculated from TGA data using Kissinger's method and activation energies used to fit PCFC results

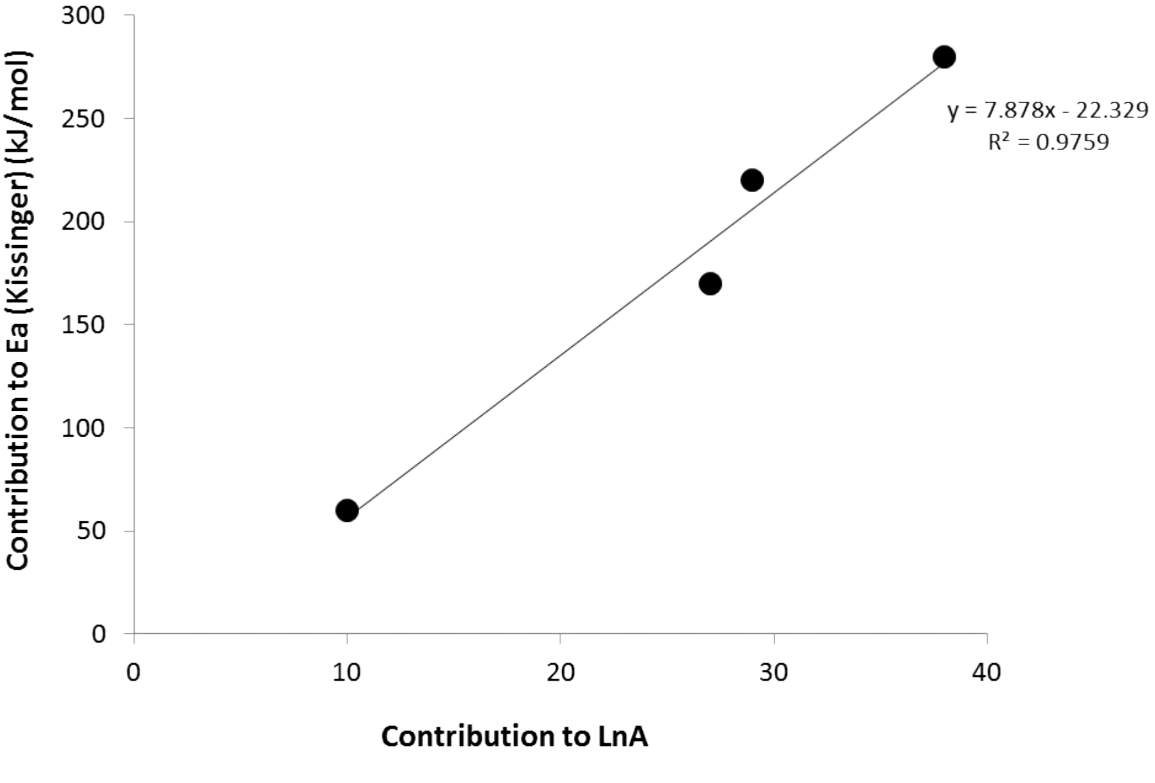

Figure S2 – Correlation between the contributions to activation energy and frequency factor

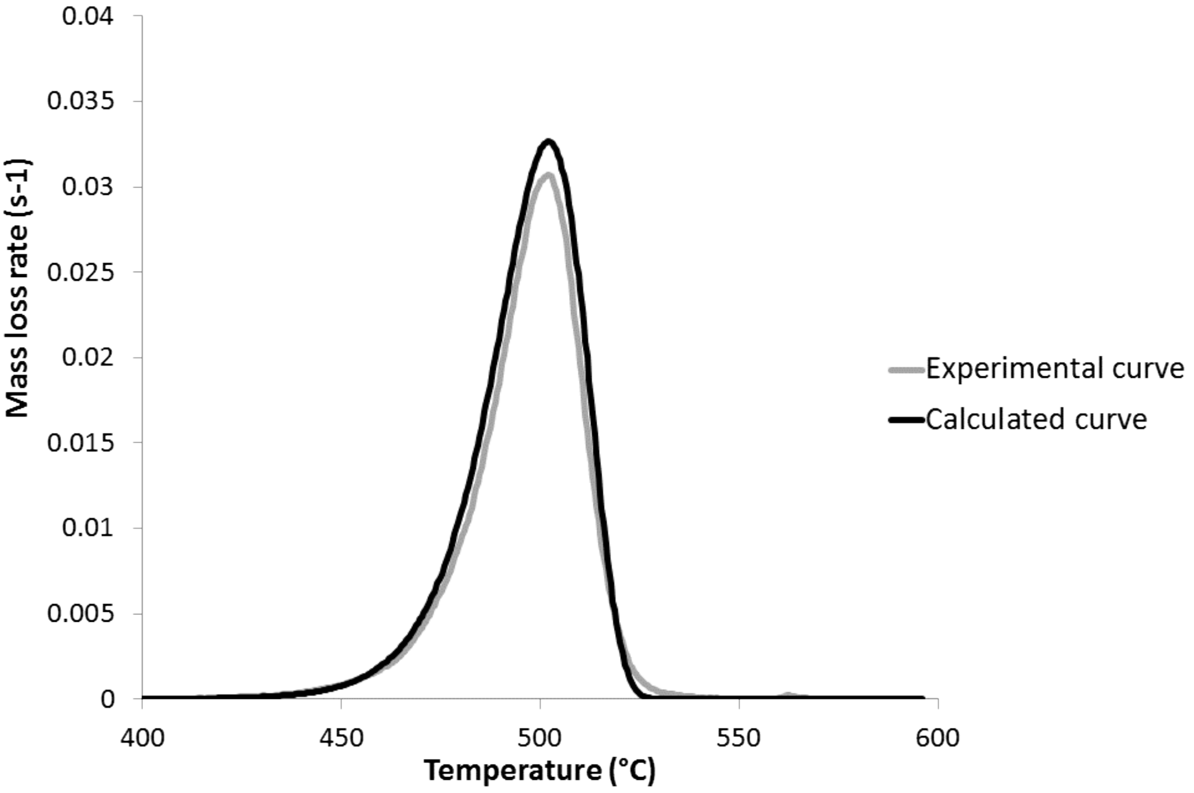

Figure S3 – Experimental versus calculated mass loss rate curves for PE at 1 K/s

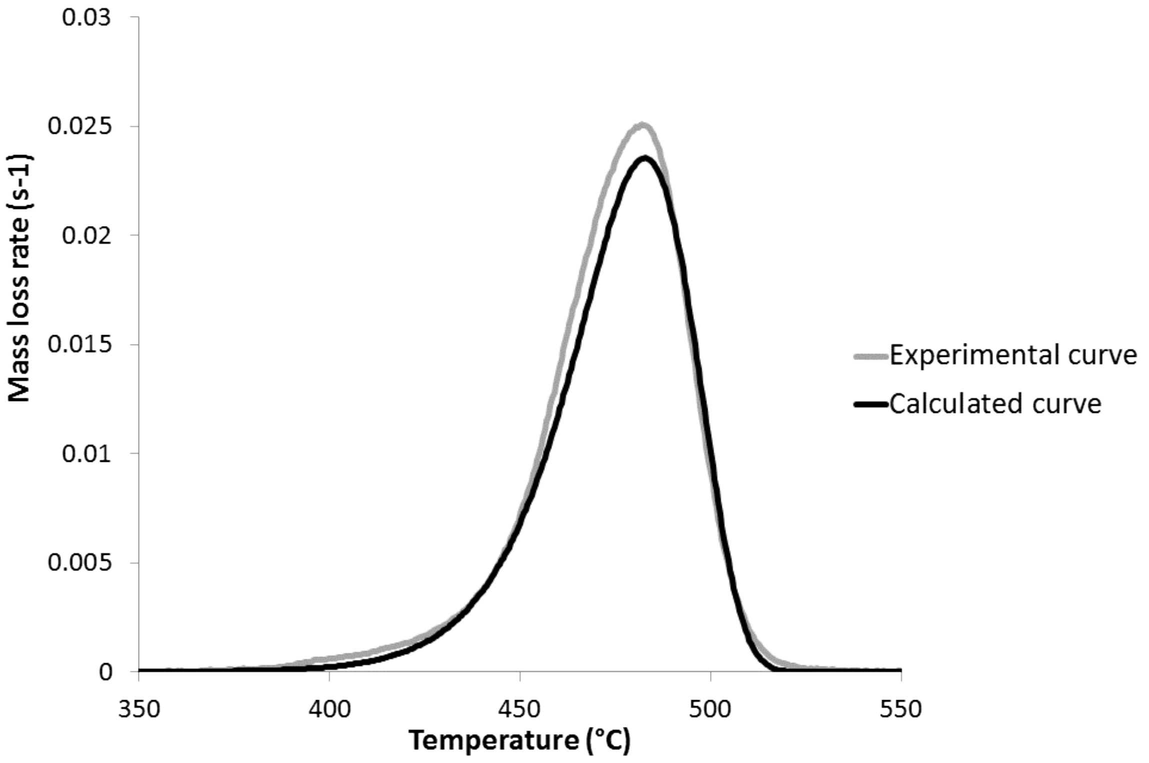

Figure S4 – Experimental versus calculated mass loss rate curves for PP at 1 K/s

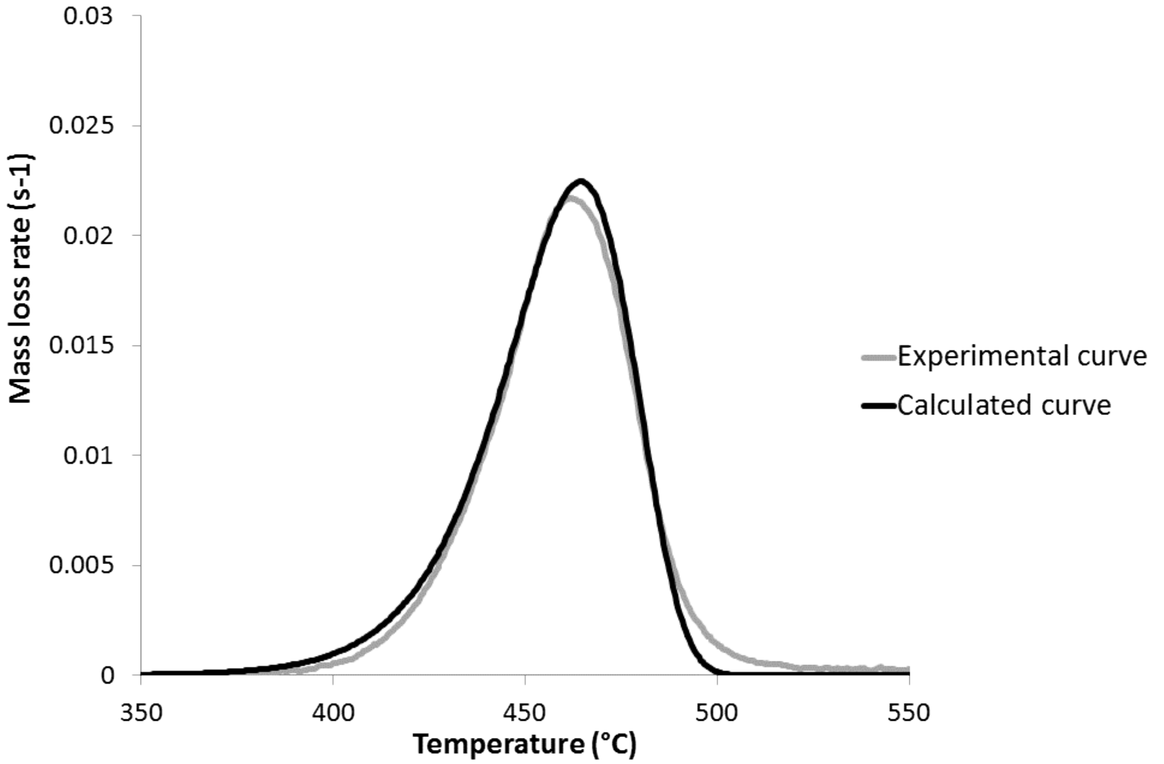

Figure S5 – Experimental versus calculated mass loss rate curves for PET at 1 K/s

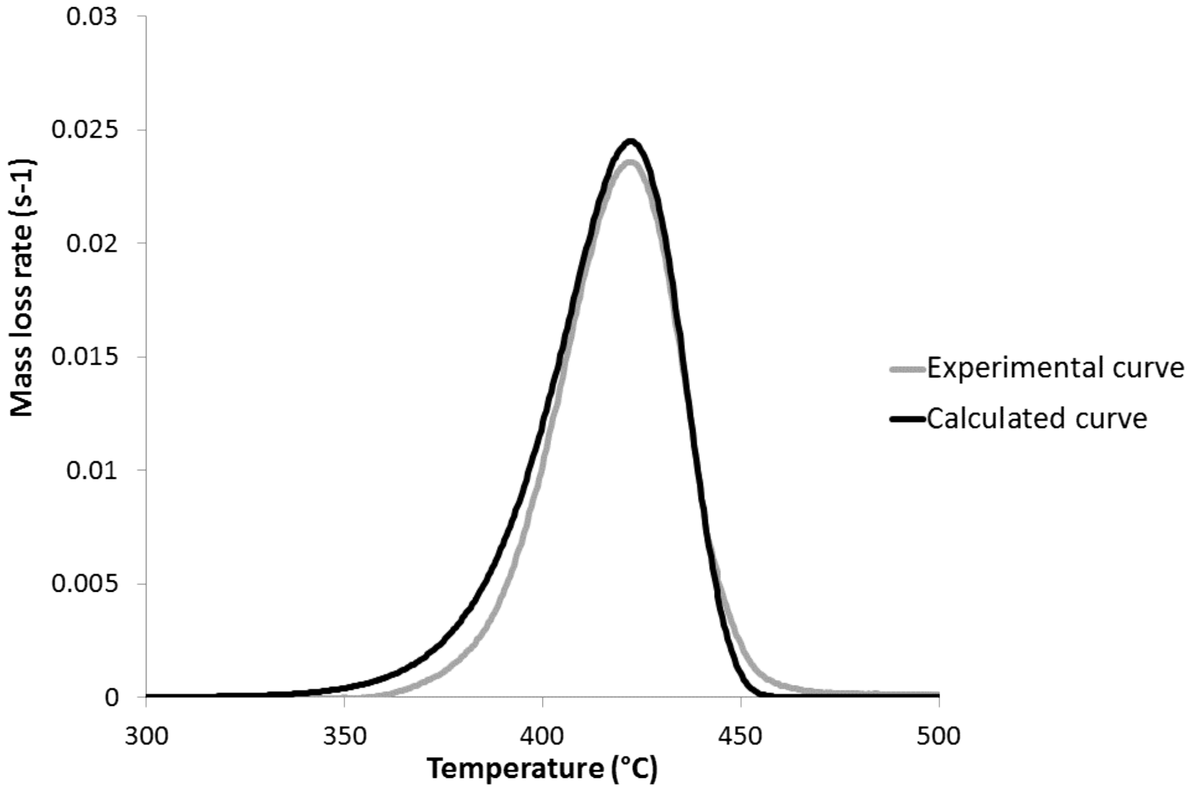

Figure S6 – Experimental versus calculated mass loss rate curves for PBT at 1 K/s

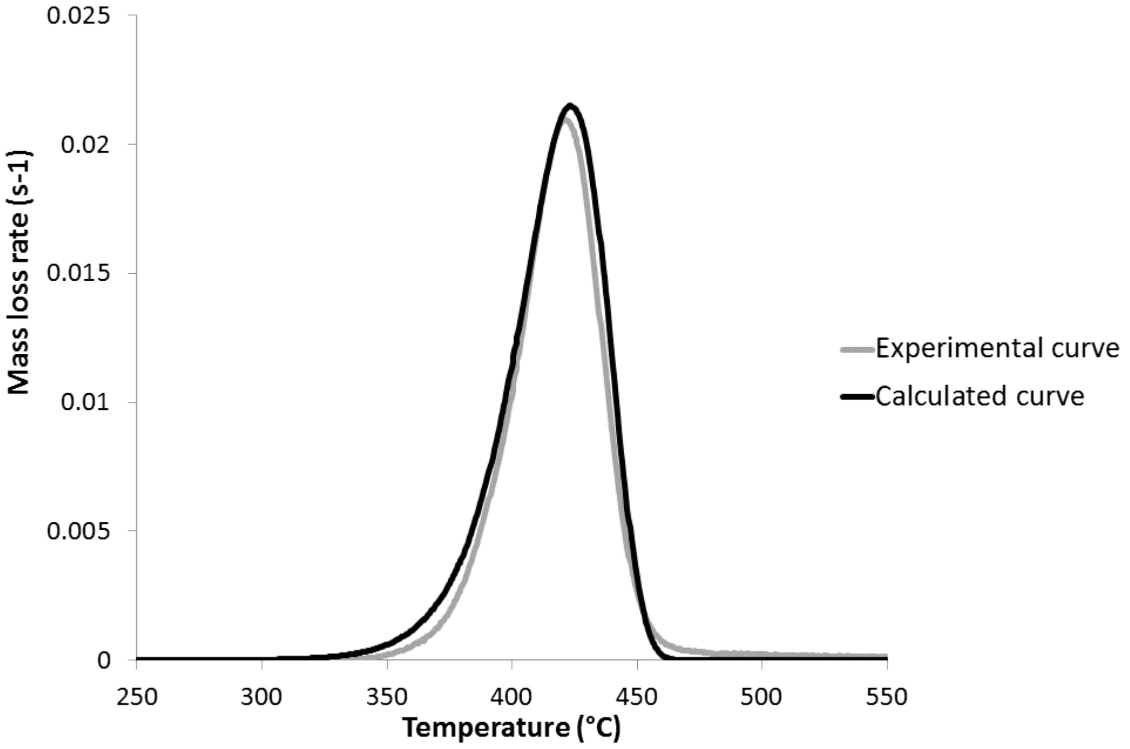

Figure S7 – Experimental versus calculated mass loss rate curves for PBS at 1 K/s

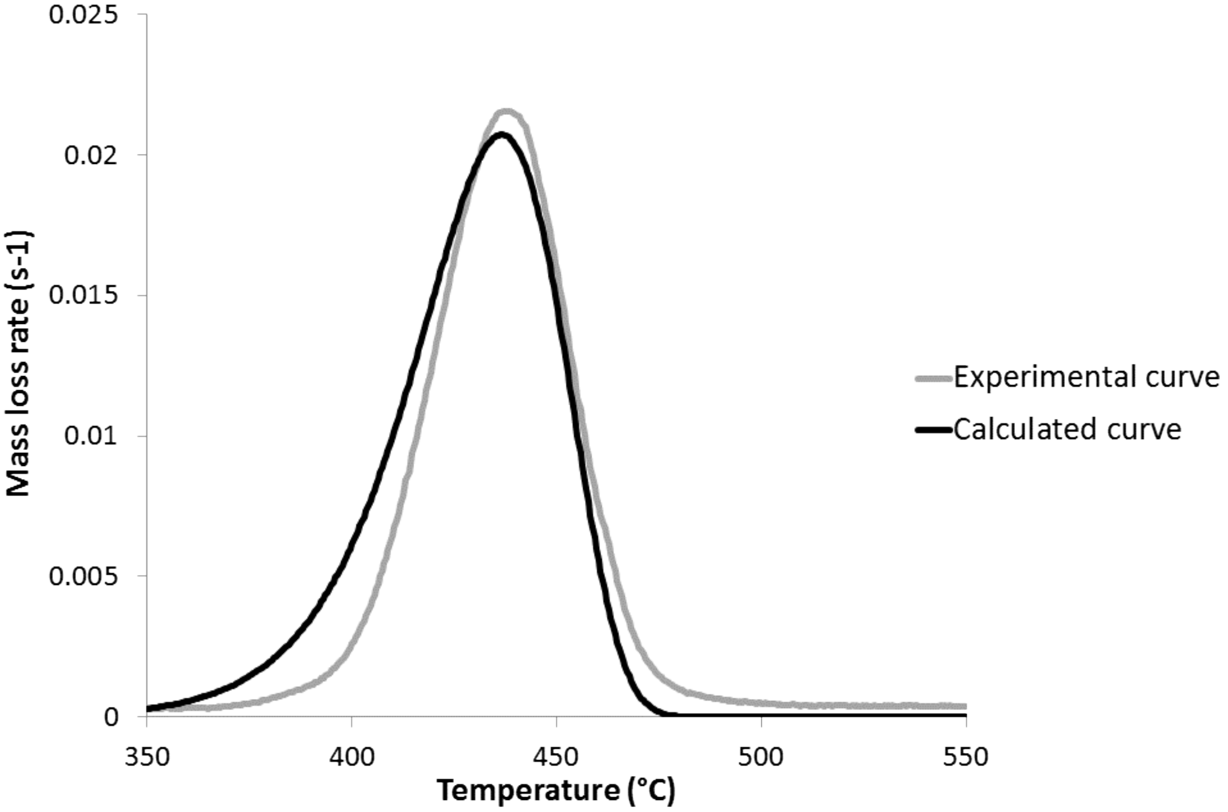

Figure S8 – Experimental versus calculated mass loss rate curves for PCL at 1 K/s

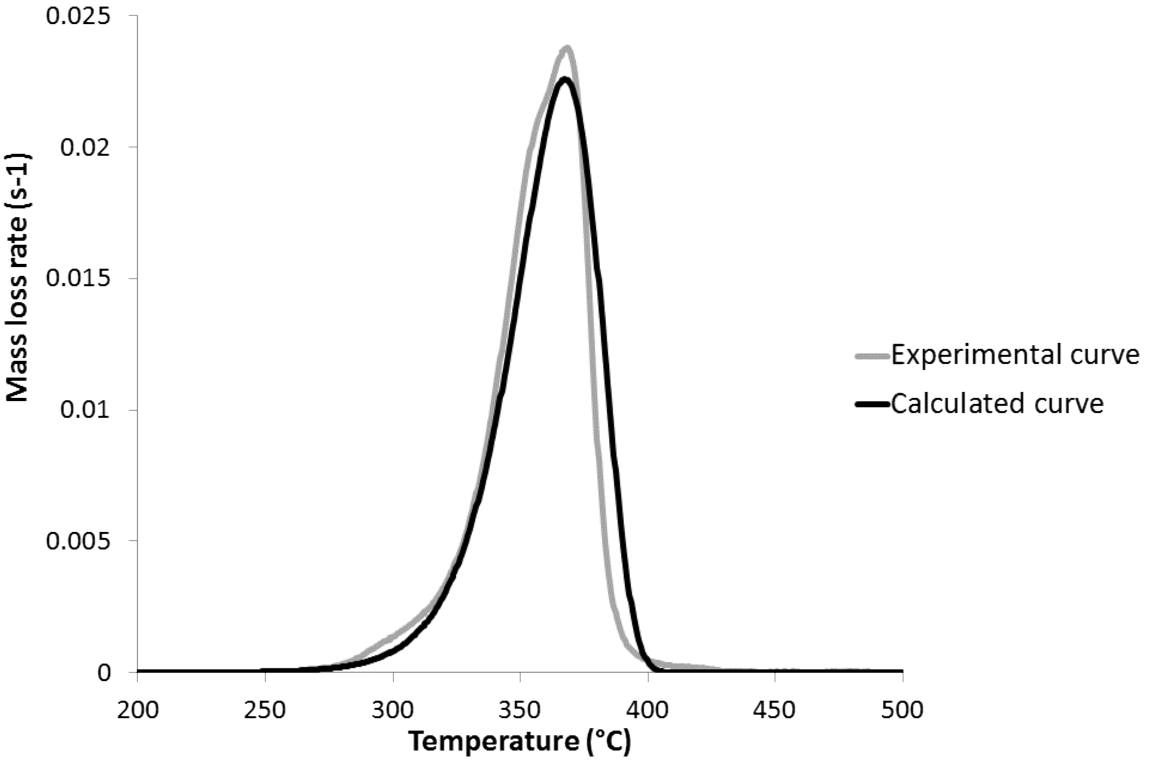

Figure S9 – Experimental versus calculated mass loss rate curves for PG at 1 K/s

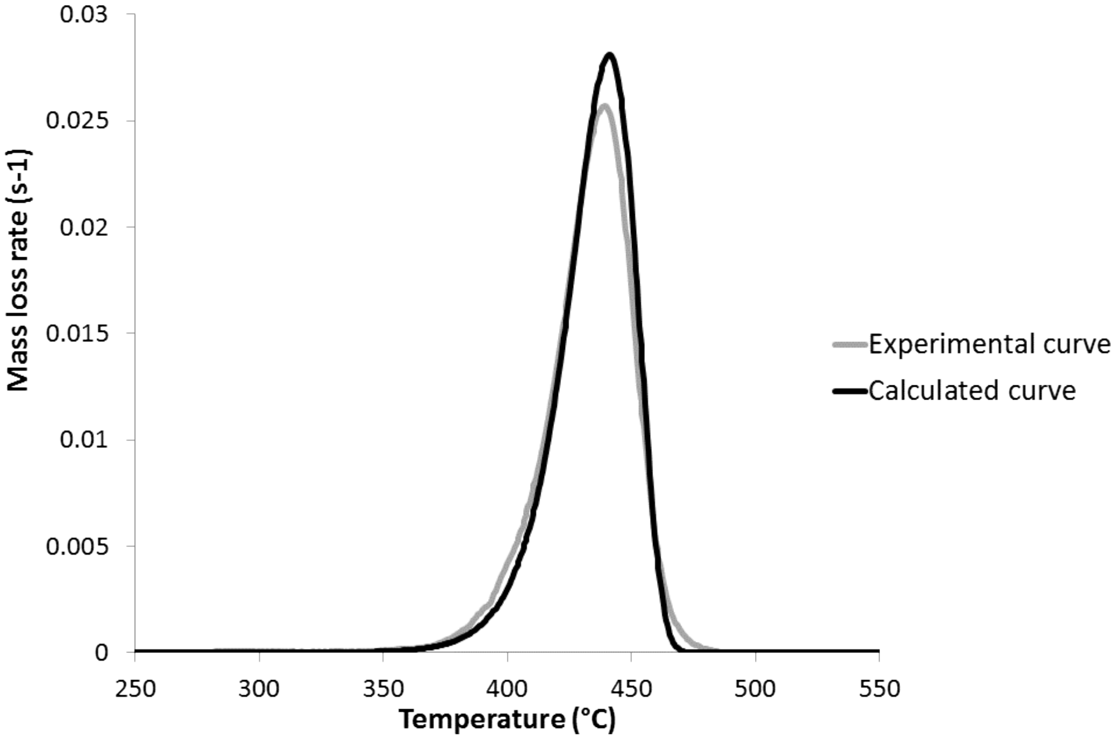

Figure S10 – Experimental versus calculated mass loss rate curves for PS at 1 K/s

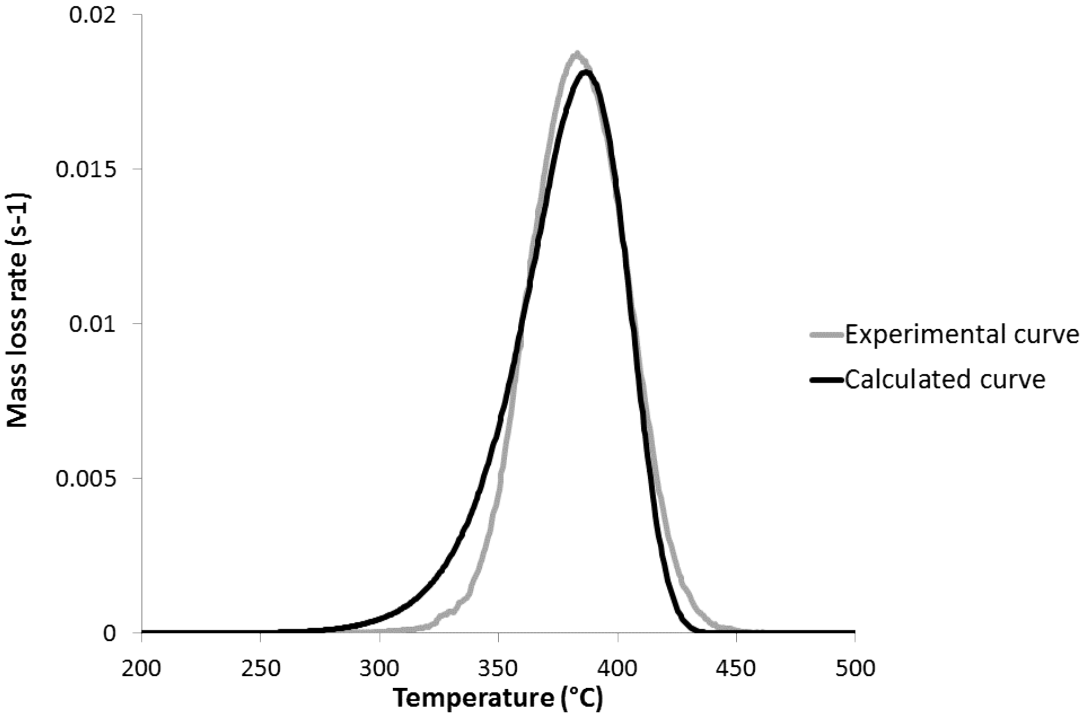

Figure S11 – Experimental versus calculated mass loss rate curves for PMMA at 1 K/s

Table S1 – Activation energies from literature for studied polymers

**Polymère**	**Flynn-Wall-Ozawa method**				**Kissinger method**	**Reference**
	**E_a (kJ/mol)**				**E_a (kJ/mol)**	
	α=0.2	α=0.4	α=0.6	α=0.8		
**HDPE**	236	248	252	250		3
**PP**	170	182	192	194		3
**PP**	176	195	202	207		8
**PP**					69	9
**PLA**	161	177	182	184		2
**PLA**					171	5
**PLA**					105	6
**PCL**	144	179	187	191		1
**PCL**					196	1
**PBS**	118	139	155	164		1
**PBS**					176	1
**PHB**	133	136	136	135		4
**PHB**					131	4
**PHB**					99	7

1 - Benarbia Abderrahim, Elidrissi Abderrahman, Aqil Mohamed, Tabaght Fatima, Tahani Abdesselam, Ouassini Krim, Kinetic Thermal Degradation of Cellulose, Polybutylene Succinate and a Green Composite: Comparative Study, World Journal of Environmental Engineering, 2015, Vol. 3, No. 4, 95-110

2 - Hantao Zou, Changhai Yi, Luoxin Wang, Hongtao Liu, Weilin Xu, Thermal degradation of poly(lactic acid) measured by thermogravimetry coupled to Fourier transform infrared spectroscopy, J Therm Anal Calorim (2009) 97:929–935

3 - A. Aboulkas, K. El harfi, A. El Bouadili, Thermal degradation behaviors of polyethylene and polypropylene. Part I: Pyrolysis kinetics and mechanisms, Energy Conversion and Management 51 (2010) 1363–1369

4 - Matko Erceg, Tonka Kova cic´ , Ivka Klaric, Dynamic thermogravimetric degradation of poly(3-hydroxybutyrate)/aliphaticearomatic copolyester blends, Polymer Degradation and Stability 90 (2005)

5 - F. Carrasco, L.A. Pérez-Maqueda, P.E. Sánchez-Jiménez, A. Perejón, Enhanced general analytical equation for the kinetics of the thermal degradation of poly(lactic acid) driven by random scission, Polymer Testing 32 (2013) 937–945

6 - Isinay E. Yuzay, Rafael Auras, Herlinda Soto-Valdez, Susan Selke, Effects of synthetic and natural zeolites on morphology and thermal, Polymer Degradation and Stability 95 (2010) 1769-1777

7 - Dimitris S. Achiliasa, Elpiniki Panayotidoua, Ioannis Zuburtikudis, Thermal degradation kinetics and isoconversional analysis of biodegradable poly(3-hydroxybutyrate)/organomodified montmorillonite nanocomposites, Thermochimica Acta 514 (2011) 58–66

8 - De-Yi Wang, Amit Das, Andreas Leuteritz, Regine Boldt, Liane Häußler, Udo Wagenknecht, Gert Heinrich, Thermal degradation behaviors of a novel nanocomposite based, Polymer Degradation and Stability 96 (2011) 285-290

9 - Xuejun Lai, Jiedong Qiu, Hongqiang Li, Rimin Zhou, Huali Xie, Xingrong Zeng, Thermal degradation and combustion behavior of novel intumescentflame retardant polypropylene with N-alkoxy hindered amine, Journal of Analytical and Applied Pyrolysis 120 (2016) 361–370
